# Visual acuity measured with luminance-modulated and contrast-modulated noise letter stimuli in young adults and adults above 50 years old

**DOI:** 10.12688/f1000research.9410.1

**Published:** 2016-08-12

**Authors:** Pui Juan Woi, Sharanjeet Kaur, Sarah J. Waugh, Mohd Izzuddin Hairol

**Affiliations:** 1 Optometry and Vision Sciences Programme, Faculty of Health Sciences, Universiti Kebangsaan Malaysia, Kuala Lumpur, 50300, Malaysia; 2 Vision and Hearing Sciences, Faculty of Science and Technology, Anglia Ruskin University, Cambridge Campus, Cambridge, CB1 1PT, UK

**Keywords:** Visual acuity, first-order, second-order, extrastriate cortex, luminance-modulated, contrast-modulated, ageing, binocular vision

## Abstract

The human visual system is sensitive in detecting objects that have different luminance level from their background, known as first-order or luminance-modulated (LM) stimuli. We are also able to detect objects that have the same mean luminance as their background, only differing in contrast (or other attributes). Such objects are known as second-order or contrast-modulated (CM), stimuli. CM stimuli are thought to be processed in higher visual areas compared to LM stimuli, and may be more susceptible to ageing. We compared visual acuities (VA) of five healthy older adults (54.0±1.83 years old) and five healthy younger adults (25.4±1.29 years old) with LM and CM letters under monocular and binocular viewing. For monocular viewing, age had no effect on VA [F(1, 8)= 2.50, *p*> 0.05]. However, there was a significant main effect of age on VA under binocular viewing [F(1, 8)= 5.67, *p*< 0.05].  Binocular VA with CM letters in younger adults was approximately two lines better than that in older adults. For LM, binocular summation ratios were similar for older (1.16±0.21) and younger (1.15±0.06) adults. For CM, younger adults had higher binocular summation ratio (1.39±0.08) compared to older adults (1.12±0.09). Binocular viewing improved VA with LM letters for both groups similarly. However, in older adults, binocular viewing did not improve VA with CM letters as much as in younger adults. This could reflect a decline of higher visual areas due to ageing process, most likely higher than V1, which may be missed if measured with luminance-based stimuli alone.

## Introduction

Visual acuity (VA) measurement is one of the clinical routines for ocular examination. VA is the capacity for seeing distinctly the details of an object. It can be described as the eye’s ability to discriminate or resolve spatially organised details. It is usually represented in two ways, which are the reciprocal of the minimum angle of resolution and Snellen fraction. The common acuity charts which are widely used are Snellen chart, logMAR chart, Kay pictures, and etc. Several conditions are known to have an impact on VA, including blur, amblyopia (lazy eye) and normal ageing ( *e.g*. Chung *et al.*, 2007; Elliot *et al.*, 1995). Clinical letter acuity charts rely on the ability of the patient to discriminate between different letters or optotypes. A common feature of letter charts is having black letters on a white background, resulting in maximum difference in luminance or brightness between them. As such, these letters can be classified as luminance-based or first order stimuli.

The human visual system is sensitive at detecting objects or images irrespective of the types of features defining them. First-order or luminance-defined information is known to be processed by linear mechanisms through linear processing within the striate visual cortex (V1). Stimuli which portray variation in properties such as contrast, texture or orientation without any change in mean luminance are known as second-order stimuli ( Hairol *et al.*, 2013; Sukumar & Waugh, 2007; Wong *et al.*, 2001). Processing mechanisms of second-order stimuli are thought to be more complex, and occur in higher and more binocular areas of the visual cortex, than those of first-order stimuli ( *e.g.* Calvert *et al.*, 2005; Hairol & Waugh, 2010; Wong *et al.*, 2005). Neurophysiology studies in cat ( Mareschal & Baker, 1998) and primates ( Baker & Mareschal, 2001) support the idea that neurons in extrastriate visual cortex, for example V2, are more responsive to contrast-modulated stimuli, compared to neurons in V1. Further evidence is shown by functional magnetic resonance imaging (fMRI) findings in human brain ( Ashida *et al.*, 2007; Larsson *et al.*, 2006).

It is well known that human visual performance reduces with normal ageing, *i.e*., ageing that is free of pathology or disease. In the past 30 years, many experimental studies have been conducted to investigate changes in a number of visual functions. Frisen & Frisen (1981) stated that VA shows a monotonic rise towards the age of 25 years and a gradual decline thereafter. The most marked decline occurs after the age of 60. Another study showed a slow but significant gradual worsening of VA after 50 years of age ( Elliot *et al.*, 1995). Spatial contrast sensitivity for first-order, luminance based gratings is also one of the visual functions which shows significant reduction in elderly compared to younger adults, even when senile miosis and reduced optical transmission factors are taken into consideration ( Elliott *et al.*, 1990). Contrast sensitivity for second-order stimuli in healthy elderly declines earlier with slower progression rate compared to that measured with first-order stimuli ( Tang & Zhou, 2009). These visual deficits in the elderly cannot be fully attributed to optical changes, but may be due to changes in retina and/or visual pathway ( Spear, 1993). Stereoacuity in healthy older adults is also reduced even without cognitive impairment such as Alzheimer’s disease ( Bassi *et al.*, 1993), implying that deterioration in binocular vision and binocular neurons of the visual cortex occurs later in life, even when VA is relatively spared. Ageing also increases contrast threshold for detecting second-order stimuli than for first-order stimuli ( Habak & Faubert, 2000). As the ageing population increases, there is a pressing need to identify the nature of perceptual capabilities in elderly. In this study, we measured and compared VA between visually healthy older and younger adults using luminance-modulated (LM) and contrast-modulated (CM) noise letters using the staircase method. This study of age-related visual system can act as a model for addressing questions relevant to a general understanding of the effects of ageing on neural information processing and VA deterioration.

## Methods

### Observers

Five older adults (mean age: 54.0±1.83 years old) and five younger adults (mean age: 25.4±1.29 years old) were recruited for the experiment. All of them underwent complete ocular health examinations to ensure that no ocular pathologies or binocular anomalies were present. None of them had any history of systemic diseases or medication with known ocular involvement. All participants wore their best refractive correction, with corrected distance VA of logMAR 0.1 (Snellen 6/7.5) or better for older adults and logMAR 0.0 (Snellen 6/6) or better for younger adults. Two sessions of training (approximately 1 hour) was made compulsory before formal data collection began to ensure that participants were familiar with the experiment. Written consent was obtained from all participants before the start of any data collection. The Ethics Committee of Faculty of Health Sciences, Universiti Kebangsaan Malaysia approved the conduct of this research (UKM 1.5.3.5/244/NN-053-2015).

### Apparatus

Stimuli were displayed on a computer screen (ViewSonic Professional Series P227f) using a custom-written program in Matlab (Mathworks, Inc) on a Dell Precision T1600 CPU. The stimuli were loaded on to the frame store memory of a VSG graphic card (Cambridge Research Systems) installed in the computer. Monitor calibration and gamma correction procedures were carried out every 3 to 6 months by using OptiCal photometer to avoid adjacent pixel nonlinearity ( Bertone *et al.*, 2011; Hairol *et al.*, 2013). In every session, the display monitor was turned on for at least 20 minutes to stabilise its luminance output before data collection commenced.

### Stimuli

**Figure 1.**
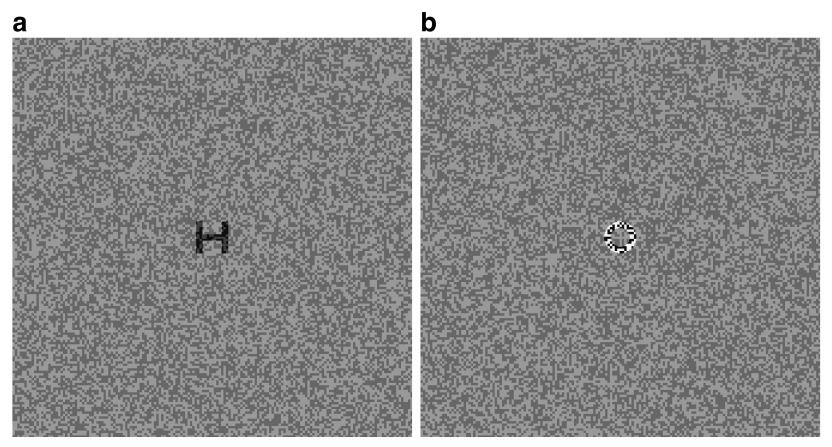
Stimuli, ( **a**) luminance-modulated (LM) letter and ( **b**) contrast-modulated (CM) letter.

Recognition of luminance-modulated (LM) and contrast-modulated (CM) letters was determined using H, O, T, and V, derived from the clinically used Sloan letters. The HOTV letters were constructed on a 5×5 template, where each stroke of the letter is one fifth of the letter’s size. The LM letters (an example shown in Figure 1a) were created by adding a luminance modulation function to a binary white noise carrier. The CM letters (an example shown in Figure 1b) were created by multiplying a modulation function with a binary white noise carrier (eg. Chung *et al.*, 2006; Hairol *et al.*, 2013; Hairol & Waugh, 2010). The stimuli can be mathematically expressed as follows:

*I*( *x*, *y*) = *I* [1+ *nN*( *x*, *y*) + *lL*( *x*, *y*) + *mnM*( *x*, *y*) *N*( *x*, *y*)] (Equation 1)

where *I* ( *x*, *y*) is the luminance at position ( *x*, *y*); *I* is the mean luminance; *n* is the noise contrast, which was fixed at 0.2 for all experiments; *N*( *x*, *y*) is the binary noise value at position ( *x*, *y*) of −1 or 1; l is the luminance amplitude, which is zero for CM letters; *m* is the contrast amplitude, which is zero for LM letters; *L*( *x*, *y*) is the luminance modulation function, a square wave; and *M*( *x*, *y*) is the contrast modulation, also a square wave. For generation of LM and CM stimuli, either *l* or *m* was adjusted, respectively, the other being set to zero. Total size of noise matrix was 500 pixels. Noise checks were scaled to the letter size and each letter consisted of 15 noise checks with 0.47 mm pixel size for one noise check. Noise was presented dynamically throughout the experiment to avoid any luminance artefacts which may occur due to pixel clumping ( Smith & Ledgeway, 1997; Sukumar & Waugh, 2007).

### Letter resolution threshold

Letter resolution threshold was measured using staircase method with a four spatial alternative-forced-choice paradigm. This method allows relatively quick estimation of threshold. The two down, one up staircase provided threshold estimation at 70.7% correct ( Shen, 2013). Participants recorded what they saw by pressing the appropriate key on the keyboard. After two successive correct responses, the size of the letter was reduced by approximately 0.125 logMAR. An incorrect response resulted in 0.125 logMAR increase in the letter size, i.e. a reversal of the staircase. There was no time limit for stimulus presentation. Eight reversals of staircase method ended the experimental run, and acuity threshold was estimated using the last six reversals. A run consisted of 30–40 trials. Data from four runs were averaged to obtain the mean acuity threshold. The experiment was run under binocular viewing and monocular viewing. In monocular viewing, the non-dominant eye was occluded with a black patch. The viewing distance between participant and monitor screen was 9 m for LM letters (achieved with a front-surfaced mirror) and 4.5 m for CM letters. Room luminance was kept constant across the testing distance.

## Results

The mean VA (logMAR) measured with LM and CM letters of all participants are shown in Table 1. For monocular viewing, in younger adults, VA with LM letters was 1.18 × better than that in older adults while VA with CM letters was 1.24 × better than that in older adults. For binocular viewing, VA with LM letters in younger adults was 1.27 × better than that in older adults while VA with CM letters in younger adults was 1.58 × better than that in older adults.

**Table 1.** Mean VA (logMAR) with LM and CM letters for older and younger adults.

	Older adults		Younger adults	
	Monocular (Mean ± SE)	Binocular (Mean ± SE)	Monocular (Mean ± SE)	Binocular (Mean ± SE)
LM VA (logMAR)	-0.12 ± 0.06	-0.15 ± 0.07	-0.18 ± 0.04	-0.24 ± 0.04
CM VA (logMAR)	0.44 ± 0.02	0.39 ± 0.03	0.34 ± 0.05	0.20 ± 0.03

**Figure 2.**
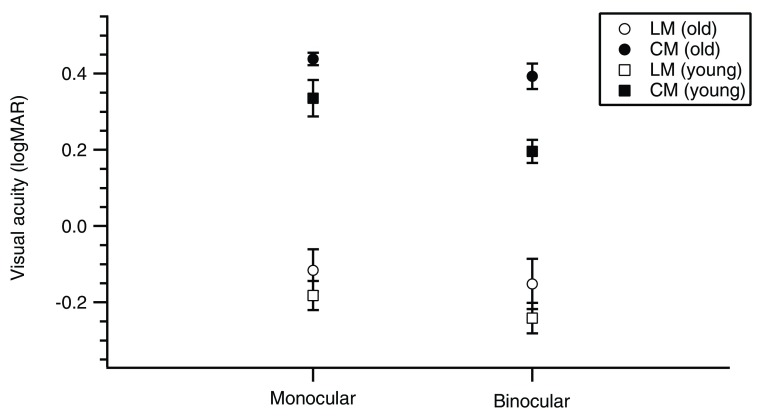
Mean VA (logMAR) for LM (open circles: younger adults; open squares: older adults) and CM (filled circles: younger adults; filled squares: older adults) letters under monocular and binocular viewing.

In Figure 2, VA of younger adults were always better than older adults’, regardless of stimulus type and viewing condition. For monocular viewing, VA with LM letters were significantly better than with CM letters in older and younger adults [F(1, 8)= 427.63, *p*< 0.001]. There was no significant main effect of age [F(1, 8)= 2.50, *p*> 0.05] on VA. There was also no significant interaction between stimulus type and age on VA [F(1, 8)= 0.50, *p*> 0.05], that is, the difference in VA in the two participant groups was similar for the two stimulus types. For binocular viewing, VA with LM letters were significantly better than with CM letters in older and younger adults [F(1, 8)= 609.58, *p*< 0.001]. However, there was a significant main effect of age on VA [F(1, 8)= 5.67, *p*< 0.05], that is, the difference in VA in the two participant groups was significantly different for the two stimulus types [F(1, 8)= 7.27, *p*< 0.05].

Table 2 shows binocular summation ratios [defined as monocular VA (MAR)÷binocular VA (MAR)] for LM and CM letters in older and younger adult groups. ANOVA test showed no main significant effect of age on stimulus types and binocular summation ratios, [F(1, 8)= 0.67, *p*> 0.05]. However, the mean difference between binocular acuities of CM letters in older and younger adults was 0.19 logMAR, which was approximately two lines worse on the letter charts and may be of clinical significance.

**Table 2.** Binocular summation ratios with LM and CM letters for older and younger adults.

	Older adults		Younger adults	
	LM letters	CM letters	LM letters	CM letters
Binocular summation ratio (Mean ± SE)	1.16 ± 0.21	1.12 ± 0.09	1.15 ± 0.06	1.39 ± 0.08

**Figure 3.**
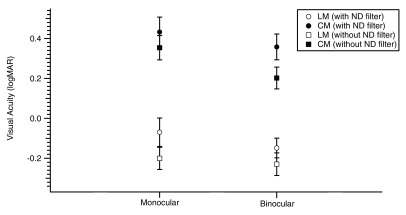
Mean VA (logMAR) with LM (open symbols) and CM letters (filled symbols) under monocular and binocular viewing for with (circles) and without neutral density (ND) filter (squares) in younger adults.

Retinal illuminance for older adults is reduced to between 10%–33% that of younger adults ( Weale, 1961). In order to simulate the reduced retinal illumination in older adults, younger participants were tested with neutral density (ND) filters. Three of the younger participants were re-tested with 85N6 ND filters (Kodak) which reduced light transmission to 19%. The stimuli and procedures were the same as described in the main experiment. VA with and without ND filters for LM and CM letters are compared in Figure 3. There was no interaction between stimulus type and retinal illumination on VA for both monocular [F(1, 2)= 1.44, *p*> 0.05] and binocular viewing [F(1, 2)= 14.85, *p*> 0.05], that is, the difference in VA for LM and CM letters measured with and without ND filters was not statistically significant.

VA with LM and CM letters in older and younger adults VA (logMAR) with LM and CM letters in older and younger adults under monocular and binocular viewing were measured. Click here for additional data file. Copyright: © 2016 Woi PJ et al. 2016 Data associated with the article are available under the terms of the Creative Commons Zero "No rights reserved" data waiver (CC0 1.0 Public domain dedication).

Binocular summation ratios with LM and CM letters in older and younger adults Monocular VA (MAR) was divided by binocular VA (MAR) to obtain the binocular summation ratios for LM and CM letters in both participant groups. Click here for additional data file. Copyright: © 2016 Woi PJ et al. 2016 Data associated with the article are available under the terms of the Creative Commons Zero "No rights reserved" data waiver (CC0 1.0 Public domain dedication).

VA for both stimulus types with and without neutral density (ND) filter in younger adults To investigate the factor of reduced retinal illumination in older adults, VA (logMAR) for LM and CM letters with ND filters under monocular and binocular viewing were measured in three of the younger participants. We compared the VA for both stimulus types with and without ND filters under monocular and binocular viewing. Click here for additional data file. Copyright: © 2016 Woi PJ et al. 2016 Data associated with the article are available under the terms of the Creative Commons Zero "No rights reserved" data waiver (CC0 1.0 Public domain dedication).

## Conclusions and discussion

VA with LM and CM letters in visually normal older and younger adults were investigated in this study. In previous psychophysical studies, VA with LM stimuli is found to be better than that with CM stimuli in normal young adults ( Hairol *et al.*, 2013; Waugh *et al.*, 2010). Similar results were shown in our study, where VA with LM letters was better than that with CM letters in visually normal older and younger adults ( Table 1 and Figure 2). The worse acuity for CM letters suggests that larger scale mechanisms are needed for CM information processing compared to that for LM, similar to the findings of Schofield & Georgeson (1999), and Sukumar & Waugh (2007).

An effect of ageing on perception of CM stimuli has been reported by Habak & Faubert (2000). They found that the contrast sensitivity for CM stimuli of older adults was significantly worse than that for LM stimuli, which is consistent with our results. Besides, our findings is in accordance with study of Tang & Zhou (2009) as well. They showed that contrast sensitivity for second-order stimuli begins to decline significantly earlier than for first order stimuli, and with a slower rate of progression. These findings suggest that CM stimulus processing mechanisms may be more vulnerable to neurophysiological changes during ageing.

A noteworthy finding in this study is that the binocular summation ratio for CM letters in younger adults was higher than that for LM letters, while the binocular summation ratio for CM letters was almost similar to LM letters in older adults. Waugh & her colleagues (2009) measured the monocular and binocular detection thresholds for LM and CM Gaussian blobs, and showed that binocular summation ratios for CM stimuli were equal or higher than that for LM Gabors for all modulation frequencies above 0.5 cycles per degree (cpd), and were more consistent across modulation frequencies. The findings led to a speculation that CM stimuli are likely to be processed in more binocular areas than LM stimuli, such as in V2, given the predominantly binocular nature of V2 neurons ( Hubel & Livingstone, 1987). Indeed, human fMRI study ( Calvert *et al.*, 2005) and psychophysical study in amblyopes ( Wong *et al.*, 2005) also suggested that CM processing may involve higher visual areas than that for LM. The lesser improvement of CM VA during binocular viewing in older adults compared to younger adults, suggests that CM stimuli neural processing mechanisms may be more vulnerable to neurophysiological changes that are associated with increasing age, may start earlier in higher visual areas. In fact, single-unit recordings showed decreases in which signal-to-noise ratio and sensitivity in cortical neurons of elderly monkeys, and these losses were even more robust in V2 than in V1 neurons ( Wang *et al.*, 2005).

Our results of binocular summation ratios for LM stimuli in younger and older adults are not consistent with the findings of Pardhan (1996). She measured monocular and binocular contrast sensitivities at spatial frequencies of one and six cpd in young and older adults with normal healthy eyes. Binocular summation ratios were higher for the younger adults compared to the older adults for both spatial frequencies. However, vertical sinusoidal gratings were used as the target for her study for determining participants’ contrast sensitivity threshold while we used noise letter stimuli for resolution threshold. The difference in binocular summation ratios between younger and older adults in Pardhan’s study could be due to the difference in stimuli and tasks.

We have shown that VA reduction in magnitude for CM letters was greater than that for LM letters under binocular viewing. However, older adults experience age-related physiological changes in vision, which included senile miosis, ie. reduction in pupil size. Senile miosis lowers retinal illumination levels, thereby affecting visual performance in elderly ( Winn *et al.*, 1994). Reduced retinal illumination in older adults may have lead to the difference between older and younger participants’ visual performance. Our retinal illumination control experiment showed no significant difference between VA for both stimulus types with and without the presence of neutral density filters. This is consistent with the study done by Habak & Faubert (2000) where they showed that contrast thresholds for LM and CM gratings are similar with or without neutral density filters on younger adults. Therefore, it appears unlikely that our findings were a result of the reduced retinal illumination in our older adults group.

In conclusion, reduction of VA for CM letters is higher than that for LM letters especially in binocular vision for healthy older adults compared to younger adults. This suggests that a young and intact visual cortex plays a key role for good visual performance with CM letters. This extra age-related VA deficit may not be fully revealed when measured with luminance-based stimuli alone. A decrease in binocular summation ratio with ageing for CM letters in older adults may reflect an early decline in higher visual areas, most likely higher than V1. This speculation is supported by the study of Costa *et al.* (2013) which suggested that ageing might have a more pronounced effect in higher visual areas than in the primary striate cortex. It is vital to know what biological (e.g. neural function, optical changes) and environmental characteristics (e.g. daily lifestyle, dietary) differentiate those older adults who lose little to no visual performance as they age and those who do not ( Owsley, 2011). It is also prudent to include a clinical measure of VA to examine age-associated differences in neural activity during cognitive processing that may affect neurologic functioning, e.g. dementia, cerebrovascular disease and depression ( Daffner *et al.*, 2013). A quick yet effective VA test is commonly being relied on to measure visual performance. However, the rate of VA changes during ageing which measure with luminance-based acuity charts may not be sufficient to be considered as ‘clinically normal’. Therefore, the advantage of CM stimuli which may serve to more sensitively detect early visual deterioration in ageing should be further investigated. A limitation in our study is that our older adults (54.0±1.83 years old) are relatively young by WHO definition ( http://www.who.int/healthinfo/survey/ageingdefnolder/en/). Therefore, a model of VA deterioration with LM and CM stimuli throughout wider normal healthy age groups is worth to be explored in future studies, which could lead to the development of a prototype of a novel CM based acuity test that might benefit the older age population.

## Data availability

The data referenced by this article are under copyright with the following copyright statement: Copyright: © 2016 Woi PJ et al.

Data associated with the article are available under the terms of the Creative Commons Zero "No rights reserved" data waiver (CC0 1.0 Public domain dedication). 

F1000Research: Dataset 1. VA with LM and CM letters in older and younger adults, 10.5256/f1000research.9410.d132585 ( Woi *et al.*, 2016a).

F1000Research: Dataset 2. Binocular summation ratios with LM and CM letters in older and younger adults, 10.5256/f1000research.9410.d132586 ( Woi *et al.*, 2016b).

F1000Research: Dataset 3. VA for both stimulus types with and without neutral density (ND) filter in younger adults, 10.5256/f1000research.9410.d132587 ( Woi *et al.*, 2016c).
