# Supplementary material for: Visual acuity measured with luminance-modulated and contrast-modulated noise letter stimuli in young adults and adults above 50 years old
Source: F1000Res. 2016 Aug 12;5:1961. [Version 1] doi: 10.12688/f1000research.9410.1 (PMC5289100; doi:10.12688/f1000research.9410.1)
Supplement: Peer review history

# Peer review: Referee response for version 1

**DOI:** 10.5256/f1000research.10134.r18036

**Published:** 2016-11-28

**Authors:** Li-Ting Tsai

**Affiliations:** 1 School of Occupational Therapy, College of Medicine, National Taiwan University, Taipei, Taiwan

This is a well written manuscript describing the effects of aging on monocular and binocular visual acuity by recognizing luminance-modulated and contrast-modulated letters. There are some points, outlined below, which need clarification or further consideration by the authors.

1. The visual acuity (Methods) for younger adults seems to be better than the older adults. Could the authors add each observer’s original data for OD, OS, and OU acuity as another supplementary data? And note which eye was used in the monocular experiment?
2. In the method session, please explain the experimental sequences of LM and CM under monocular and binocular viewing.
3. In the method session, please add the value of contrast to display the letter stimuli.
4. The additional experiment - the authors tended to use the ND filters to simulate the reduced retinal illumination in older adults due to senile miosis. However, the possible reasons or mechanism related vision changes in older adult are not limited to senile miosis or higher visual areas. In fact, the visual acuity of older adults seems to be lower than those of younger group. However, it is suggested to discuss other possible reasons to contribute the results.

I have read this submission. I believe that I have an appropriate level of expertise to confirm that it is of an acceptable scientific standard.

# Peer review: Referee response for version 1

**DOI:** 10.5256/f1000research.10134.r16356

**Published:** 2016-09-16

**Authors:** Wan Hazabbah Wan Hitam

**Affiliations:** 1 Department of Ophthalmology, University of Science, Malaysia, Kubang Kerian, Malaysia

Overall, this is a good study adding a new knowledge to the field.

 Comment and suggestions:

- The title is very interesting. 
- The abstract does not contain clear objective of the article written. The conclusion also is not clearly stated.
- There are no keywords written.
- The introduction is good and the objective is stated nicely.
- The methods section is overall acceptable. However, the sample size calculation is not mentioned. The number of sample collected is rather small.
- The results presented are satisfactory. Analysis done is appropriate.
- Overall the discussion is good. The limitation of the study should be addressed clearly.
- The conclusion is too long. It should answer the objective. 
- The number of references is adequate and updated
- The Figures and Tables are satisfactory.

I have read this submission. I believe that I have an appropriate level of expertise to confirm that it is of an acceptable scientific standard.

# Peer review: Referee response for version 1

**DOI:** 10.5256/f1000research.10134.r15649

**Published:** 2016-08-25

**Authors:** Goro Maehara

**Affiliations:** 1 Department of Human Sciences, Kanagawa University, Yokohama, Japan

The authors examined the effect of aging on visual acuity measured using the luminance-modulated and contrast-modulated letter stimuli. The main claim of the paper is that the advantage of binocular viewing (binocular summation) was smaller for the older adults than for the younger adults when the contrast-modulated stimuli were presented. Although their aim is of scientific and clinical interests, the results are not clear enough to draw any strong conclusion. Comments are detailed below.

 Major Concerns:

 1. The main experiment

 Visual acuity was generally better for binocular viewing than for monocular viewing. The younger adults showed better visual acuity than the older adults. These results are clear but not surprising. Although the interactions among age, stimulus types, and viewing conditions are important here, they were too small to draw any conclusions. The authors reported that there were the significant interactions. However, I guess that they conducted the two-way ANOVAs separately for binocular and monocular viewing. This is not appropriate. The authors should conduct the three-way (age **× **stimulus type **× **viewing condition) ANOVA. This ANOVA yielded no significant interaction. I encourage the authors to increase the number of observers for making meaningful conclusions because there were only 5 observers for each age group.

 2. The additional experiment

 It can be seen from Figure 1 and 3 that visual acuity was comparable between the older adults and the younger adults who viewed the stimuli through a ND filter. This suggests that the reduction in retinal illumination caused the differences between the age groups. Please analyze and discuss this comparison.

 The authors argued that the age-group differences cannot be attributed to the illumination reduction because there was no significant interaction between stimulus type and retinal illumination. However, only 3 observers participated in the experiment. Their argument is far from convincing. It is necessary to increase the number of observers. Please conduct the three-way ANOVA for this experiment, too.

 Minor concerns:

 1. Please state clearly what statistical analyses were conducted.

 2. Typo in page 3. “artcfacts” should be “artifacts”.

I have read this submission. I believe that I have an appropriate level of expertise to confirm that it is of an acceptable scientific standard, however I have significant reservations, as outlined above.
